# Together for Health: An Initiative to Access Health Services for the Hispanic/Mexican Population Living in the United States

**DOI:** 10.3389/fpubh.2019.00273

**Published:** 2019-09-24

**Authors:** María Gudelia Rangel Gómez, Ana María López Jaramillo, Alejandro Svarch, Josana Tonda, Juanita Lara, Elizabeth J. Anderson, Cecilia Rosales

**Affiliations:** ^1^Department of Population Studies, El Colegio de la Frontera Norte, Tijuana, Mexico; ^2^US-Mexico Border Health Commission, Tijuana, Mexico; ^3^Secretariat of Health, Mexico, Mexico; ^4^Institute of Mexicans Abroad, Mexico, Mexico; ^5^Division of Public Health Practice & Translational Research, University of Arizona Mel & Enid Zuckerman College of Public Health, Tucson, AZ, United States

**Keywords:** access to health services, preventive programs, collaborative programs, migration and health, mobile health services, immigrant

## Abstract

A disproportionately small percentage of the Hispanic/Mexican population in the United States has adequate access to health services, which decreases quality of life at both the individual and community levels. In addition, it increases risk for preventable diseases through insufficient screening and management. The Mexican Section of the U.S./Mexico Border Health Commission, in efforts to address barriers to accessing preventive health care services for vulnerable populations, launched the initiative *Juntos por la Salud* (JPLS) that offers health promotion and disease prevention services to Hispanics living in and around 11 U.S. metropolitan cities via mobile health units. This paper presents a descriptive analysis of the JPLS initiative and potential positive impact it has had in reducing barriers faced by the Hispanic population. JPLS screens and provides referrals to primary care services to establish a medical home and has the potential to reduce health care costs in a high-risk population through education and timely health screenings.

## Introduction

The health system in the United States (U.S.) divides the population among those who receive public or private medical care and those who cannot access health services (1). This only serves to heighten health disparities and create structural barriers to accessing available and affordable health care services (2). Moreover, lack of access to health services for undocumented residents in the U.S. affects both physical and emotional health (3, 4).

Despite the implementation of the Patient Protection and Affordable Care Act (ACA), the number of vulnerable Mexican immigrants with deteriorating health situation continues to grow. The ACA was established through federal legislation in 2010 to address high uninsurance rates, lack of access to care, and variable quality of care received in the U.S. (5). Immigration status often plays a role in lack of access to health insurance and quality health services. However, in some states, special programs are available for vulnerable populations, specifically women and children. California, for example, offers MEDI-CAL and New York offers Child Health Plus, a health insurance plan for children (6). Federally Qualified Community Health Centers (FQHCs) are also available and affordable for those with limited resources and lacking health insurance, regardless of immigration status.

According to studies carried out by the Mexican *Consejo Nacional de Población* (CONAPO-Nacional Council on the Population of Mexico), about 6.2 million Mexican immigrants (more than 50% of this population) did not have health insurance or access to health services between 2004 and 2013. Recent data indicate that 45% of undocumented immigrants are currently uninsured whereas only 8% of citizens are uninsured; in families with mixed citizenship statuses, children with one or more non-citizen parent are twice as likely to be uninsured than children of citizens (7). This proportion is higher compared to other groups of immigrants (8); however, this figure did decrease (3%) in the indicated period (6). This slight decrease could be attributed to the passage of the ACA, which increased coverage for all populations, but most notably for Hispanics. While the uninsured rate for all Hispanic adults was 40.5% prior to the main provisions of the ACA taking effect in 2013, this rate decreased slightly by 7.1 percentage points in 2014 (9).

CONAPO data also demonstrated that males of Mexican origin living in the U.S. were less likely to have health insurance coverage than their female counterparts in 2013, when 55% of the Mexican immigrant population with insurance coverage was female. Only one in three youth between 18 and 29 years of age were covered by health insurance. Further, four out of ten children under 18 years of age, six out of ten adults between 30 and 44 years of age and half of adults between 45 and 64 years of age were without health insurance (10).

Barriers to access to health services for Mexican migrants in the U.S. include socioeconomic factors such as low educational attainment, and working in low-paying occupations (11–14), which may be related to underemployment, undocumented employment, or low knowledge about worker's rights. Race and ethnicity are additionally associated with reduced access to offers of employment-based health insurance for minority groups including Hispanics compared to white workers in similar occupations (15). In addition, immigration status or immigration status of family members are associated with reduced utilization of health care services due to fears of discrimination or legal repercussions (16). Commonly cited as barriers to seeking basic health care include lack of understanding of the U.S. healthcare system, language barriers (4, 17–21), and work- related migration between the U.S. and Mexico as well as within the U.S. (22–25).

Given limited health care utilization by Mexican immigrants to the U.S., the government of Mexico launched an initiative called the *Ventanillas de Salud* (VDS; “Health Windows”) in 2003. The mission was improving access to primary and preventive health services, increasing public insurance coverage, connecting individuals to medical homes, and promoting a culture of self-care in Mexicans living in the U.S. Currently, the Mexican Consulates in the U.S. operate 49 VDS and two mobile VDS. In addition to general health information, the VDS provide: counseling and guidance services about disease prevention and health promotion; preventive health screening; referral to primary healthcare services; and eligibility aids for ACA insurance plans.

From 2013 to 2017, the VDS provided 21 million services to 7.7 million people. In the same period, the number of preventive health screenings offered grew by 337% and the number of Mexican immigrants qualifying for health insurance in the U.S. increased by 106% (26). This increase demonstrates both direct and indirect contributions of the VDS to the health and well-being of the Mexican immigrant population and in keeping with the overarching mission of decreasing emergency room visits and uncompensated care.

### *Juntos por la salud* Initiative

A diagnostic health mapping of the Mexican population living in the U.S. conducted by the Mexican Section of the US/Mexico Border Health Commission (CSFMEU) found that the state of health of a large proportion of the Hispanic population was gravely vulnerable, especially in geographic areas beyond the reach of the VDS. Identified risk factors for vulnerable health status included immigration status, limited English proficiency, low economic status, and few or no services in remote areas where people reside, all of which contribute to limited or no access to health services (4). Additionally, the political context of the U.S. regarding migrants is associated with increased uncertainty and stress (16) which are barriers to accessing both social and health services by the population most in need.

Thus, VDS expansion was deemed necessary to reduce health related risks for the most vulnerable subsets of Hispanics in the U.S. CSFMEU introduced the *Juntos por la Salud* Mobile Health and Wellness Initiative (JPLS). The aim of this initiative was to strengthen the VDS strategy and provide preventive health services to rural and hard-to-reach communities in the U.S. with difficult access to regular medical care. JPLS is an ongoing collaboration between the Mexican Ministry of Health (SSA), the Office of Foreign Relations (SRE), the Institute of Mexicans Abroad (IME), and the *Ventanillas de Salud* (VDS) Strategy. JPLS mobile units differ from mobile VDS services because they operate independently from the consulates and are organized at the community level.

The primary goal of JPLS is to reduce barriers to health care for the Mexican population residing in the U.S. by providing access to preventive programs in their preferred language and in a culturally sensitive and linguistically appropriate manner. However, the reach and potential for public health impact created by JPLS has not been previously described in the scientific literature. The objective of this paper is to present a qualitative descriptive analysis of the JPLS initiative and its potential to reduce barriers faced by the Mexican immigrant population living in the U.S. JPLS is hypothesized to increase access to health care services and encourage establishment of a medical home, thus reducing health care costs through education and timely health screenings.

**Figure 1:**
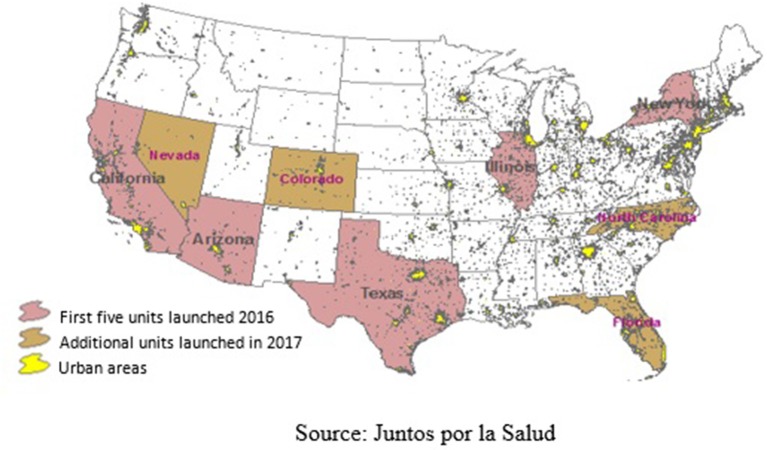
Geographic locations of *Juntos por la Salud* Mobile Health Units.

In 2016, the initial phase was launched with five mobile health units in the following cities: Chicago (Illinois), Dallas (Texas), Los Angeles (California), Phoenix (Arizona), and New York (New York). Subsequently, in a second phase, an additional six mobile health units launched in the cities of Denver (Colorado), Las Vegas (Nevada), Tucson (Arizona), Miami (Florida), Orlando (Florida), and Raleigh (North Carolina) (Figure 1).

JPLS provides health education on priority health issues, such as nutrition, obesity, diabetes, women's health, children's health, mental health, substance use, exposure to violence, HIV/AIDS and other sexually transmitted infections, as well as legal and financial guidance. In addition, mobile health units provide preventive health screenings, referrals to clinics or community programs, follow-up on referrals and administration of immunizations. All participants are informed and verbally consent to share their personal information and risk factors. Each mobile health unit has built a network of local health agencies and community based organizations increasing access to a number and diversity of health care services, as well as referrals to local community clinics at low cost or free of charge. All of the services at JPLS are provided free of charge regardless of national origin.

Each mobile unit is managed and operated by a leading local agency in the U.S. and has a network of strategic allies integrated by specialized health institutions in the area including hospitals, laboratories, community clinics, and community programs that coordinate to handle referrals. All of the materials offered at the JPLS units are developed to be culturally appropriate and are offered by bilingual staff and volunteers. Staffing is highly dependent on the fiscal agent operating the mobile health unit and includes community health workers, health science students, nurse assistants, or public health trained professionals. Mobile unit locations are determined monthly based on neighborhood demography (i.e., concentration of vulnerable Hispanics/Mexicans) and invitations from strategic allies per their agency policies. Locations and hours of services vary and are primarily advertised via social networks, local radio stations and word-of-mouth.

Additional activities of the individual mobile health units included: participatory relationship-building with existing health and community stakeholders; monitoring of high-risk cases; implementation of capacity-building programs to train health workers among residents of targeted communities; registration and monitoring of the services offered; and maintenance of the mobile health units. Program goals included the following, though data related to their success are not reported: refer ~50% of un- or under-insured users to appropriate public benefit health insurance programs; provide medical referrals to 100% of individuals with abnormal health screening results to a primary care provider; establish a medical home for ~50% of clients referred to community clinics; provide general health consumer education sessions to individuals at the mobile health units during the term of this contract.

## Methods

### Data Collection at JPLS Mobile Health Units

JPLS uses a database system which captures information on those served through JPLS and services provided, for the period between February 2016 and December 2018. The JPLS mobile health units were strategically placed in target cities based on the concentration of Hispanic population determined by the network of consular offices in the U.S. and the Mexico Section of the U.S. Mexico Border Health Commission. Additional health and needs assessment were subsequently conducted by each community based organization funded within the 11 mobile health unit sites selected.

### Descriptive Analysis

Our descriptive analyses are derived from fully anonymized deidentified secondary data sources with no individual-level identifiers. Demographic and epidemiological characteristics of the target population were summarized from an ecological level (i.e., only population summary data are available). Prevalence estimates for commonly identified disease states and count data for health screening, health education and referral services provided through JPLS were estimated. As this analysis used secondary data and was not deemed human subjects research, ethical approval was not required.

## Results

### Services Provided

**Figure 2:**
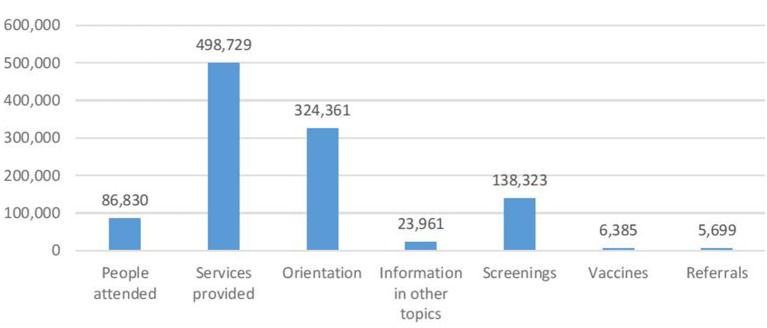
*Juntos por la Salud* Program: services provided (February 2016–December 2018). *Juntos por la Salud*, CSFMEU.

Between February 2016 and December 2018, JPLS registered 86,830 participants and provided 498,729 services including health education, preventive health screenings, vaccination, and referrals, among others. On average, each participant received 5.7 services during each visit (Figure 2). Of over 86,000 service recipients in the mobile units, 86% reported not having medical insurance.

Of the total services provided, 65% involved health education on priority health issues, raising awareness about the importance of timely detection related to chronic diseases, oral health, eye care and occupational health; nutrition and physical activity, sexual and reproductive health, and mental health. Approximately, 30% of total services included the following preventive health screenings: blood pressure, glucose levels and hemoglobin A1c, anthropometric measurements, cholesterol, and HIV/AIDS.

Other services offered by these mobile health units include group health education sessions on topics such as prevention of chronic degenerative diseases, sexual and reproductive health, preventive programs (nutrition and physical activity, dental, occupational, mental or visual health), substance abuse or domestic violence, financial wellness, educational or access to secondary prevention services. Likewise, these group education sessions also hosted vaccination days to immunize against seasonal flu. During this same period, there were ~1,025 group sessions with an average of 38 attendees per session. Fifty-four percent of the education imparted included information about preventing chronic-degenerative diseases such as hypertension, diabetes, obesity and cancer, among others. Twelve percent focused on other preventive programs; nine percent sexual and reproductive health information, seven percent substance abuse, seven percent mental health information.

Those who screened positive for health conditions of interest were referred to community clinics or specialized medical services. Referrals were most frequently made for hypertension, obesity and high glucose levels, indicating chronic health problems of the targeted population. Between February 2016 and December 2018, 6,385 vaccines were administered, of which 70% were influenza vaccines and almost 20% were for Hepatitis; the remaining proportion include vaccines for tetanus, pertussis, and routine childhood immunizations.

### Demographic Profile of Participants Served

Between February 2016 and December 2018, 77% of service recipients were of Mexican origin; 7.4% were born in the U.S. and 9.8% came from Central America, South America and the Caribbean. Sixty-two percent were women and a large percentage of these women (58%) were of reproductive age. Eighty-four percent had lived in the U.S. for more than 10 years. About 29% had between 1 and 6 years of education. The three most common occupations reported by service recipients were construction, house/office cleaning and factory work. Only 20% reported English language proficiency.

### Epidemiologic Profile

The population served by JPLS from February 2016 to December 2018 indicated that 75% were overweight or obese, 31% had high cholesterol levels, and 29% had high blood pressure levels (Table 1). Moreover, prevalence of overweight and obesity in the population attended by the JPLS mobile health units was 50% higher than the population seen in the VDS, which reported a prevalence of 50%, during the period from 2013 to 2018 (26). In regards to mental health 2,389 individuals reported having a prior diagnosis of depression or anxiety, 38% reported not receiving treatment.

**Table 1:** Prevalence of the main causes of morbidity among Mexicans in the U.S. *Juntos por la Salud* initiative (February 2016–December 2018).

**Type of detection**	**Number of tests performed**	**High/positive results**	**Prevalence**
Overweight/obesity	21,110	15,721	74.5%
Total cholesterol	8,171	2,565	31.4%
Blood pressure	30,190	8,693	28.8%
Glucose	22,129	4,516	20.4%
HIV/AIDS	1,105	6	0.5%

*Juntos por la Salud data base, CSFMEU, February 2016–December 2018*.

## Discussion

The JPLS program provides needed services for a very vulnerable population in the U.S. Barriers such as their immigration status, limited proficiency of English, low economic status, and living in remote areas, yield insufficient access to health care services (9, 13). Of the total number of people served by mobile health units, 86% reported not having health insurance. This highlights their vulnerability, especially when 80% report living in the U.S. for >10 years. As for the main causes of morbidity of this population, 75% are overweight and obese, which is 25% higher than the prevalence reported by the population served by the *Ventanillas de Salud* (50%) (26). This situation indicates that the mobile health units are likely reaching individuals who are otherwise unable to access medical care and emphasizes the potential impact of the JPLS program.

Mobile health units are designed to implement screening measures that can deter serious illnesses requiring urgent medical attention, thus reducing inappropriate or inefficient medical service utilization. The need for targeted services among those who do not have consistent access to primary or preventive care is demonstrated by the higher ecological prevalence of overweight and obesity among service recipients attending the mobile clinics compared to those accessing similar services via the VDS initiative (50 vs. 75%). However, additional differences in the characteristics of those seeking care at the mobile health units, such as untreated diabetes, hypertension, or other chronic conditions are not known due to limitations in the available data. Future research on JPLS service recipients is needed to determine motivating factors for seeking care at mobile health units.

Some illnesses may be attributed to lifestyle behaviors (diet, physical inactivity, etc.), which may have changed upon arriving to the U.S. The services provided by mobile health units play an important role, especially in orientation and counseling on priority health issues, and timely detection, before experiencing a need for emergency services.

The rate of referrals to outside services by JPLS is numerically low (5,699 referrals made to 86,830 people seen, 6.5%); however, qualitatively this rate is acceptable for the purposes of the mobile unit considering the scope of work and personalized attention each referral entails. Comprehensive preventative health services that led to referrals for follow up care include: guidance and personalized counseling, navigation in accessing health services, health screenings, follow-up to establish a medical home, and assurance that the participant accessed the medical attention needed. The vast majority (86%) of the JPLS program participants reported not having any health insurance due to sociodemographic barriers to accessing health services. A central goal of the JPLS program is to connect community members with a trusted and safe health promotion environment. The 5,699 referrals were provided to individuals who have rarely or never received comprehensive services and access to care due to financial or structural barriers. However, not everyone who is offered a referral will accept or follow the referral instructions, and the acceptance rate following a JPLS visit is not currently known. Each JPLS mobile health unit is continually working to increase the number of referrals using different mechanisms that include innovative strategies to disseminate vital health information, logistics related to the referrals and building a network of trusted health institutions to link participants with services.

### Impact of the JPLS Initiative

The JPLS mobile health units purport to improve the health conditions of a Hispanic population experiencing a state of vulnerability and increased barriers to health services. However, the long-term impact of JPLS, if any, is unknown. However, we propose the following potential mechanisms for impact that warrant specific study:

- By means of health promotion and disease prevention services, JPLS is postulated to reduce emergency room visits for non-emergency health care.
- JPLS reaches the Mexican immigrant population living in the in a state of vulnerability and outside the range of the VDS (which is broadly utilized where it has been implemented).
- JPLS mobile units strengthen the efforts of VDS to reduce the prevalence of the main causes of mortality in hard-to-reach populations.
- The services provided are anticipated to be highly acceptable to the target population as they increase the network of interprofessional allies to facilitate access to health services, at low cost or free, in a linguistically and culturally appropriate manner.
- Though JPLS targets the Mexican immigrant population, the mobile health units provide service to anyone interested in them, regardless of the country of origin or insurance status.

Our initial qualitative descriptive analysis indicates a need for future exploration of the contribution of the JPLS initiative as well as a model to duplicate for greater impact and intervention studies in health care.

## Conclusion

The Hispanic population in the U.S., particularly Mexican immigrants, lack access to health care services, affecting the quality of their physical and mental well-being (26). Fear of deportation and discrimination greatly affects the population and affects social and health service-seeking behaviors. Thus, it is imperative to develop and invest in effective strategies that allow provision of basic and preventive health services such as screenings, health promotion, and disease prevention, despite the current political context in the U.S. Innovative mobile healthcare strategies such as JPLS are one potential avenue for accessing hard to reach populations including Mexican immigrants in the U.S. who may otherwise go without basic access to preventive health screening, health education, and referral services.

## Data Availability Statement

The datasets generated for this study are available on reasonable request to the corresponding author.

## Author Contributions

AL assisted with the initial proposal of the article and with the analysis of information. CR, JT, JL, EA, AS, and MR helped with the review of information, data interpretation, and discussion.

### Conflict of Interest

The authors declare that the research was conducted in the absence of any commercial or financial relationships that could be construed as a potential conflict of interest.
